# Comb-referenced laser distance interferometer for industrial nanotechnology

**DOI:** 10.1038/srep31770

**Published:** 2016-08-25

**Authors:** Yoon-Soo Jang, Guochao Wang, Sangwon Hyun, Hyun Jay Kang, Byung Jae Chun, Young-Jin Kim, Seung-Woo Kim

**Affiliations:** 1 Department of Mechanical Engineering, Korea Advanced Institute of Science and Technology (KAIST), Science Town, Daejeon, 305-701, South Korea; 2 Department of Instrument Science and Technology, College of Mechatronic Engineering and Automation, National University of Defence Technology (NUDT), Hunan, Changsha, 410073, China

## Abstract

A prototype laser distance interferometer is demonstrated by incorporating the frequency comb of a femtosecond laser for mass-production of optoelectronic devices such as flat panel displays and solar cell devices. This comb-referenced interferometer uses four different wavelengths simultaneously to enable absolute distance measurement with the capability of comprehensive evaluation of the measurement stability and uncertainty. The measurement result reveals that the stability reaches 3.4 nm for a 3.8 m distance at 1.0 s averaging, which further reduces to 0.57 nm at 100 s averaging with a fractional stability of 1.5 × 10^−10^. The uncertainty is estimated to be in a 10^−8^ level when distance is measured in air due to the inevitable ambiguity in estimating the refractive index, but it can be enhanced to a 10^−10^ level in vacuum.

Laser distance interferometers have been playing key roles for the advance of precision machines for large-volume manufacture of nanotechnology-based products such as semiconductor integrated circuits, flat panel displays and solar photovoltaic panels^1^,^2^,^3^. Such interferometers rely on continuous-wave lasers, measuring distance or length by accumulating the interferometric phase with sub-nanometer resolutions uninterruptedly over ranges up to several meters^4^. The laser wavelength used for distance measurement is calibrated to standard lasers stabilized to atomic absorption lines as recommended by the Bureau International des Poids et Mesures (BIPM) for realization of the current definition of the meter^5^,^6^,^7^. Recently the frequency comb of mode-locked femtosecond lasers (hereafter simply referred to as ‘comb’) began to be adopted as a new wavelength ruler, permitting calibration of the laser wavelength with direct traceability to the radio-frequency standard^8^,^9^ or an optical clock^10^. In addition, the comb can be used for simultaneous generation of multiple arbitrary wavelengths; by phase-locking tunable laser diodes individually to selected optical modes within the comb^11^ or by filtering out several optical modes separately with power amplification by injection-locking^12^. This approach of comb-referenced wavelength generation offers excellent frequency stability and uncertainty, allowing for implementation of advanced techniques such as absolute distance measurement by multi-wavelength interferometry^13^,^14^,^15^,^16^,^17^,^18^ and real-time compensation of the refractive index of air by two-colour interferometry^19^,^20^.

In fact, during the past decade, many attempts have been made to use the comb as the light source for long-distance measurements with various progressive principles; inter-mode synthetic wavelength interferometry^21^,^22^,^23^, many-wavelength dispersive interferometry^24^,^25^,^26^,^27^, multi-wavelength interferometry^13^,^14^,^15^,^16^,^17^,^18^, dual-comb frequency-down-conversion interferometry^28^,^29^,^30^,^31^, and optical cross-correlation time-of-flight measurement^32^,^33^,^34^. These newly proposed methods proved effective in extending the non-ambiguity distance range by taking advantage of the comb’s unique spectral or temporal characteristics. Nonetheless, for practical use in precision positioning and dimensional measurement, they need to be appraised systematically in terms of the stability and uncertainty in consideration of all possible error sources.

Our work described here is concerned with the method of comb-referenced multi-wavelength interferometry. A prototype interferometer system is used to demonstrate its competence as an absolute-type positioning transducer. As illustrated in Fig. 1, the interferometer employs four different wavelengths concurrently phase-locked to the comb of an Er-doped fiber laser. The absolute position is determined through synchronized phase detection along with parallel data-processing of the multiple interference phases so as to enable fast, precise and stable measurements continuously over a few meters of axis-travel. In fact, the interferometer system was previously proposed in ref. 18 by the authors together with primary measurement results. In this study, with further experimental data obtained via more elaborate environment control, comprehensive analysis is made to validate the proposed interferometer system as a practical means of realizing the definition of the meter with an utmost level of uncertainty and stability.

## Results

### System configuration

**Figure 2:**
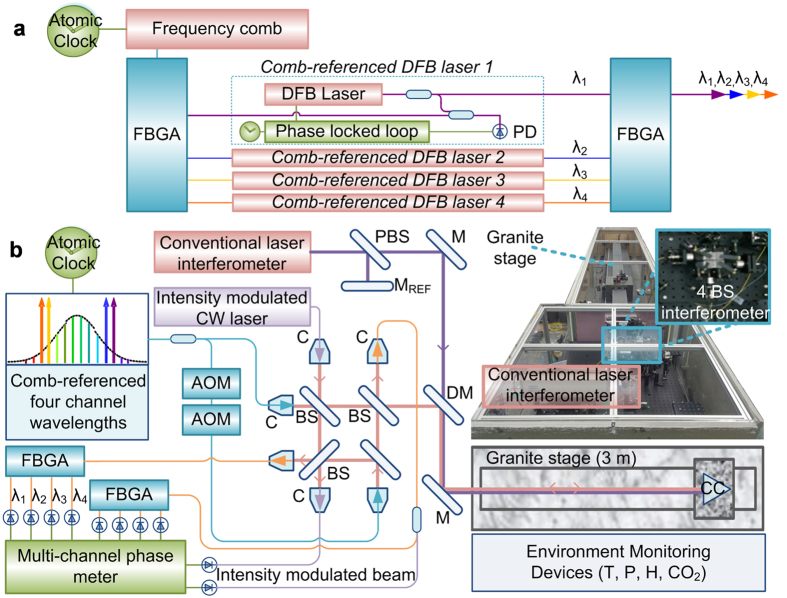
Interferometer system configuration. (**a)** Comb-referenced four wavelength light source. (**b)** Hardware design of the comb-referenced multi-wavelength interferometer. Abbreviations are; FBGA: fiber Bragg grating array, OFG: optical frequency generation, DFB: distributed feedback laser, PD: photo-detector, AOM: acousto-optic modulator, C: collimator, BS: beam splitter, M: mirror, DM: dichroic mirror, CC: corner cube, T: temperature, P: pressure, H: humidity and CO_2_: carbon dioxide concentration.

Figure 2 illustrates the comb-referenced multi-wavelength interferometer system constructed in this study. The light source comprises four distributed feedback (DFB) diode lasers, each being phase-locked to a distinct wavelength within the comb of an Er-doped fiber femtosecond laser of a 1550 nm center wavelength. The comb used as the wavelength ruler is stabilized to the Rb atomic clock by phase-locked loop (PLL) control of the repetition rate (*f*_*r*_) precisely to 100 MHz and also the carrier-envelop-offset frequency (*f*_*ceo*_) to 30 MHz. The *f*_*ceo*_ is extracted using a fiber-type *f*-*2f* interferometer^35^,^36^. The four DFB laser output beams, each having an optical power of 10 mW, are combined into a single-mode fiber so that they propagate along the same optical path together all the way within the interferometer. For heterodyne interferometric phase detection, the combined DFB lasers are divided into two fibers, one of which is connected to a pair of acousto-optic modulator (AOM) to provide a frequency-shift of 40 kHz. The configured interferometer optics is basically of Michelson type, employing four non-polarizing beam splitters so as to divide and also recombine the reference and measurement beams without the polarization leakage causing non-linearity errors. Once the original and frequency-shifted beams have been recombined after round-trip to the reference and measurement arms, the DFB lasers are separated using fiber-Bragg-grating (FBG) arrays so that the interferometric phase of each laser wavelength is detected using a multi-channel phase meter of 0.1° resolution^18^.

The light source, in addition to the four DFB lasers, incorporates a continuous-wave (CW) laser of a 1530 nm wavelength. This near-infrared CW laser is intensity-modulated with a 40 MHz rf frequency to produce a long synthetic wavelength of 7.50 m. This extra source is used to perform coarse phase-measuring interferometry to provide an initial estimate of the target distance needed for the process of multi-wavelength interferometry. In addition, for comparison purpose, a commercial HeNe laser interferometer of incremental distance measurement is installed via a dichroic mirror along the same optical path of the multi-wavelength interferometer. The whole interferometer system is shielded to prevent drastic temperature variation, while being set up on a granite frame seated on a passive anti-vibration foundation. The target mirror is a corner-cube type mounted on an aerostatic-bearing stage moving along the granite guideway. By monitoring the ambient parameters of the air temperature, relative humidity, air pressure and CO_2_ concentration, the refractive index of air is estimated for real-time wavelength compensation of the DFB lasers using the Ciddor’s formula^37^.

### Wavelength generation

**Figure 3:**
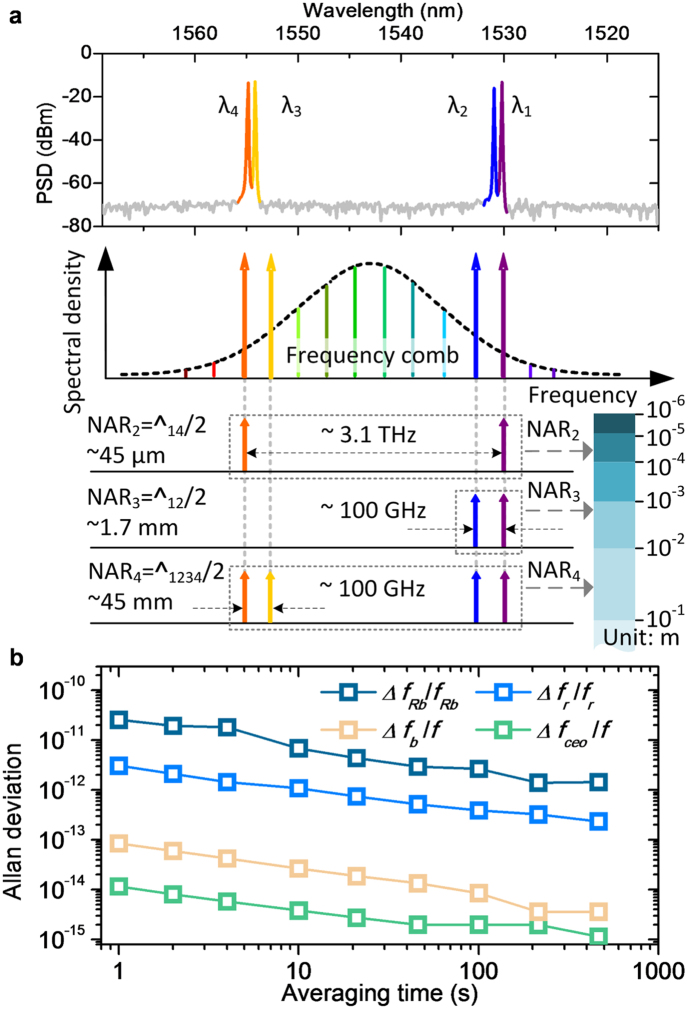
Four-wavelength-combined light source. (**a**) Measured optical spectrum and estimation of non-ambiguity range (NAR) of the combined light source. (**b)** Frequency stability measurement result.

For proper implementation of multi-wavelength interferometry, the four wavelengths employed as the light source have to be carefully selected. For convenience, the wavelengths in terms of their vacuum values are denoted as λ_*i*_ with *i* being 1, 2, 3, 4 and λ_1_ < λ_2_ < λ_3_ < λ_4_. As depicted in Fig. 3a, the selection rule adopted in this study is to maximize the non-ambiguity range (NAR) that is half of the synthetic wavelength Λ_1234_ defined by the equation of 1/Λ_1234_ = |1/Λ_12_ − 1/Λ_34_| = |(1/λ_1_ − 1/λ_2_) − (1/λ_3_ − 1/λ_4_)|. In order to make Λ_1234_ large, the separation between λ_1_ and λ_2_, and also between λ_3_ and λ_4_ are narrowed. Besides, λ_1_ and λ_4_ are placed far apart so that an intermediate synthetic wavelength Λ_14_ is created in between Λ_12_ and λ_1_ or λ_4_. The comb spectrum used as the reference in this study for wavelength stabilization has a 100 MHz mode-to-mode spacing, but the permissible mini-mum frequency separation is practically limited by the 100 GHz channel spacing of the FBGAs used for wavelength separation and recombination. Accordingly, λ_1_ and λ_2_ are located at 1530.279693 nm and 1531.040888 nm, generating a synthetic wavelength Λ_12_ of ~3.4 mm. Similarly, λ_3_ and λ_4_ are placed in the higher wavelength end of the comb spectrum, at 1554.179409 nm and 1554.937151 nm, respectively. In consequence, as illustrated in Fig. 3a, the resulting NAR extension can explained in three steps: First, the far-separated λ_1_ and λ_4_ makes a synthetic wavelength Λ_14_ = λ_1_λ_4_/(λ_1_ − λ_4_) being worked out to be 90 μm. Second, the closely located pair of λ_1_ and λ_2_ creates a longer synthetic wavelength Λ_12_ of 3.4 mm. The other pair of λ_3_ and λ_4_ yields a similar synthetic wavelength Λ_34_ of ~3.4 mm. Third, combing all four wavelengths leads to the largest synthetic wavelength Λ_1234_ of 90 mm, offering an overall NAR of 45 mm.

Figure 3a shows the optical spectrum of the light source comprising the four wavelengths actually produced with a signal-to-noise ratio of 50 dB in this study. As already explained, the comb was stabilized to the Rb clock by PLL control of the repetition rate (*f*_*r*_) and the carrier-envelop-offset frequency (*f*_*ceo*_). The optical frequency generated for each wavelength is represented by *f* = *c*/λ with *c* being the speed of light in vacuum. After the comb-referenced stabilization, the frequency can be expressed as *f* = *N* × *f*_*r*_ + *f*_*ceo*_ + *f*_*b*_ in which *N* is a large integer and *f*_*b*_ denotes the beat frequency with the optical mode designated within the comb^11^. The expression leads to the first-order frequency variation Δ*f* in a fractional form of Δ*f*/*f* = [(Δ*f*_*Rb*_/*f*_*Rb*_)^2^ + (Δ*f*_*r*_/*f*_*r*_)^2^ + (Δ*f*_*ceo*_/*f*)^2^ + (Δ*f*_*b*_/*f*)^2^]^1/2^ with *f*_*Rb*_ being the Rb atomic clock frequency. Figure 3b shows the temporal stability of each frequency term actually measured in terms of the Allan deviation with increasing the averaging time from 1 to 500 s. Note that the Δ*f*_*Rb*_ was measured by monitoring the beat signal of the Rb clock in use with another Rb clock of the same kind. Other Δ*f*_*r*_, Δ*f*_*ceo*_ and Δ*f*_*b*_ were monitored using a frequency counter referenced to the Rb clock. The stability test result indicates that the Δ*f*_*Rb*_/*f*_*Rb*_ term is the most dominant instability factor, being 2.57 × 10^−11^ at 1 s averaging. All other terms, Δ*f*_*r*_/*f*_*r*_, Δ*f*_*ceo*_/*f* and Δ*f*_*b*_/*f*, are found smaller than Δ*f*_*Rb*_/*f*_*Rb*_, making no significant contributions to the overall stability of Δ*f*/*f*. Since Δ*f*/*f* = Δλ/λ, the fractional stability obtained in frequency is directly transferred to the fractional stability in wavelength. Note that the frequency instability causes random errors, influencing the measurement repeatability. Complete estimation of the frequency uncertainty demands that not only the random frequency stability but also the systematic frequency offset of the Rb clock be taken into consideration together as will be discussed in detail later.

### Absolute distance determination

For each wavelength λ_*i*_, the refractive index of air *n*_*i*_ is estimated using the Ciddor’s equation in this study. Hence, the target distance *L* can be expressed as *L*_*i*_ = (λ_*i*_/2*n*_*i*_)(*m*_*i*_ + *e*_*i*_) with *m*_*i*_ and *e*_*i*_ being an integer and an excess fraction, i.e. 0 ≤ *e*_*i*_ < 1, respectively. The excess fraction *e*_*i*_ is determined one by one directly from the interferometric phase measured for each λ_*i*_. Next, the integer *m*_i_ is decided so as to satisfy the inequality constraint of |*L* − *L*_*i*_| < α(λ_*i*_/2) for all λ_*i*_ simultaneously with α being a positive constant selected as small as 1/100. This process is implemented in real time by numerical iteration using LabVIEW software (National Instruments) by adopting the conventional algorithm of the excess fraction method^38^, or smart algorithms devised to minimize the computation time in dealing with a large group of *m*_i_ candidates^39^,^40^. Convergence to a unique true solution of *m*_*i*_ requires that the uncertainty of *L*_*i*_ should be less than a quarter of λ_*i*_, i.e. Δ*L*_*i*_ < λ_*i*_/4. Otherwise, the numerical computation would fail to converge at all or reach a false solution that deviates from the true solution with a large error. If abnormal convergence happens, the calculated integer value of *m*_i_ shows a drastic change from its previous value. This situation can be ruled out using a simple criterion. i.e. |Δ*m*_*i*_| between two consecutive calculations should be either 0 or 1 for every λ_*i*_.

**Figure 4:**
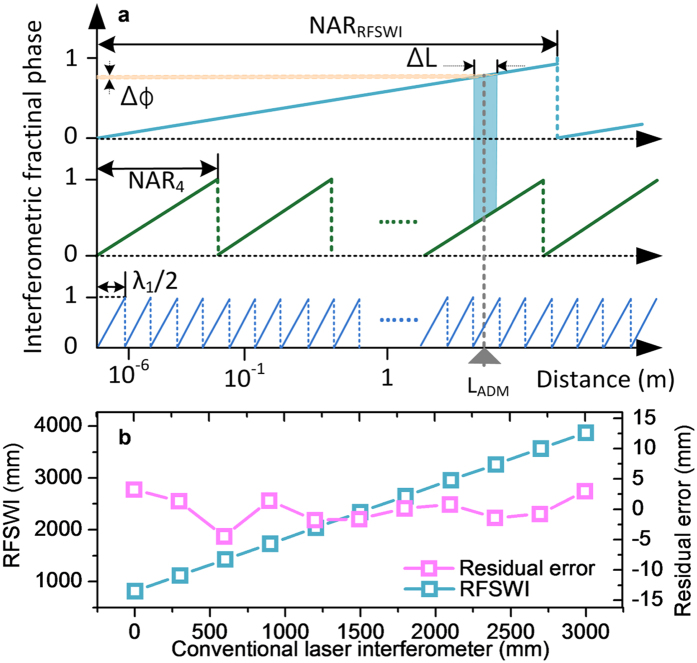
Non-ambiguity range (NAR) extension with radio-frequency synthetic wavelength interferometry (RFSWI). (**a)** Synthetic wavelength chain with uncertainty requirement. (**b)** Comparative measurement result between the RFSWI and cw HeNe laser interferometer.

Beyond the non-ambiguity range (NAR_4_) of 45 mm, the measurement range is extended by operating the cw laser added in the light source. The cw laser is intensity-modulated with a 40 MHz frequency so that radio-frequency synthetic wavelength interferometry (RFSWI) is implemented with a prolonged non-ambiguity range (NAR_RFSWI_) of 3.75 m as illustrated in Fig. 4. The RFSWI permits coarse measurement of *L*, which is subsequently taken as the initial guess of *L* for the numerical computation of multi-wavelength interferometry to accurately determine *m*_*i*_ for each λ_*i*_. The NAR extension demands that the RFSWI measurement uncertainty be less than a half of NAR_4_, i.e. <22.5 mm over the entire range of NAR_RFSWI_. This uncertainty requirement was confirmed using the HeNe laser interferometer installed in parallel with the multi-wavelength light source as explained in Fig. 2, revealing that the RFSWI offers an accuracy of 7.7 mm in peak-to-valley as presented in Fig. 4b.

### Measurement results

**Figure 5:**
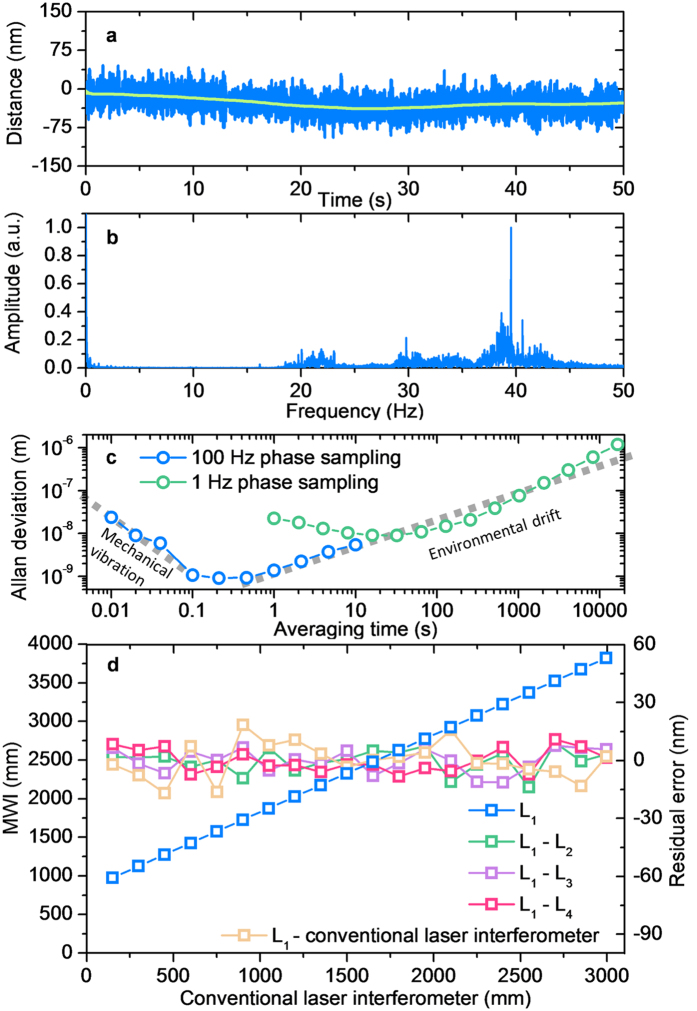
Performance test result. (**a)** Time-dependent variation of the measured distance *L*_1_ over 50 s. (**b)** Fourier-transformed data of *L*_1_. (**c)** Allan deviation of *L*_1_. (**d)** Linearity comparison with an incremental HeNe laser interferometer. Inter-comparisons between individual distances of four wavelengths are also shown.

Figure 5 presents a test result in which the comb-referenced multi-wavelength interferometer built in this study was operated from 1.0 to 3.8 m in steps of 150 mm. At each step, the stage was brought to a complete stop on the granite guideway; the motion control servo and also the aerostatic bearing were turned off so that distance readings were taken without disturbance from servo-induced motion jitters or turbulence-induced bearing vibration. Figure 5a shows a typical time-dependent variation of the measured distance of *L*_1_ from λ_1_ over a 50 s period monitored at an updated 100 Hz rate. The stage holding the target mirror was positioned at a 3.8 m distance. The standard deviation of the repeated measurements of *L*_1_ was 20 nm, while the moving average of 100 consecutive measurements yields a peak-to-valley variation of 42 nm (standard deviation: 7.8 nm). The *L*_1_ measurement data is Fourier-transformed as shown in Fig. 5b; the high frequency fluctuation seen in the 20–40 Hz range is estimated to arise from random mechanical vibration existing on the granite guideway, while the low frequency disturbance below 1 Hz is attributable to the slow environmental drift of the refractive index of air and also the thermal expansion of the granite guideway due to the ambient temperature change.

Further, the effect of random noise on the measurement stability is analysed by calculating the Allan deviation of the measurement data as a function of the averaging time as plotted in Fig. 5c; the measurement stability, or repeatability, improves better than 1 nm as the averaging time increases from 0.01 to 1.0 s. With further increasing the averaging time, the measurement stability begins to worsen in proportion because the slow varying environmental drift becomes accumulated while fast varying noise is cancelled out. It is also noted in Fig. 5c that when the distance update rate is slowed down from 100 Hz to 1 Hz, the measurement stability worsens, by an order of magnitude, due to ineffective suppression of high frequency random noise. Lastly, Fig. 5d shows a comparison of the distance *L*_1_ with the accumulated distance of the HeNe laser interferometer over the absolute distance range of 1.0–3.8 m. Note that the HeNe laser interferometer reading has a zero-datum offset of ~1.00 m from the absolute distance reading. The comparison was made at every 150 mm step of the stage movement by averaging 300 measurements taken at a 100 Hz update rate. The result reveals a peak-to-valley discrepancy of 35.3 nm, which corresponds to a ±4.6 × 10^−9^ linearity error between the two interferometers over an entire range of 3.0 m. Figure 5(d) also shows comparisons between absolute distances of *L*_1_, *L*_2_, *L*_3_ and *L*_4_ obtained the from multi-wavelength interferometer. With distance differences of *L*_1_ − *L*_2_, *L*_1_ − *L*_3_ and *L*_1_ − *L*_4_ lying within a 20 nm peak-to-valley bound, the maximum linearity error is worked out to be ±2.6 × 10^−9^ between the measured distances from individual wavelengths. The inter-wavelength differences are reckoned to arise from the imperfect compensation of the refractive index of the ambient air due to the 10^−8^ level uncertainty of the Ciddor’s equation formula.

**Figure 6:**
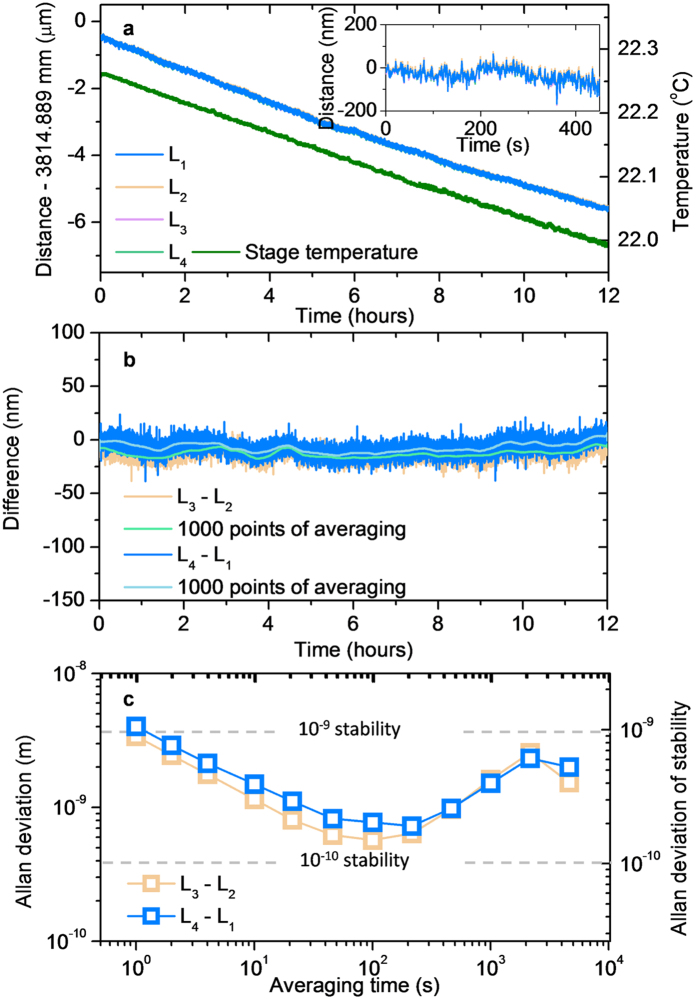
Long-term test result. (**a)** Time-dependent variation of the distances measured from the four wavelengths along with the stage temperature change over 12 hours. (**b)** Distance differences between four wavelengths. (**c**) Allan deviation of distance differences between wavelengths.

Figure 6 shows another test in which the measured distance was monitored over a long period of 12 hours at a 1 Hz update rate. The stage holding the target mirror was positioned stationary at the farthest end of a 3.8 m distance throughout the test measurement. When the absolute distances *L*_1_, *L*_2_, *L*_3_ and *L*_4_ are plotted together, they all appear to follow a common long-term variation of gradual decrease by ~5 μm. The distance decrease was caused by a temperature change of 0.3 °C that was monitored during the entire test period as in Fig. 6a. Despite the temperature variation, the individual distances of *L*_1_, *L*_2_, *L*_3_ and *L*_4_ shows no significant difference, so they are not well distinguishable from each other as plotted in Fig. 6b. The inter-wavelength distance differences of *L*_3_ − *L*_2_ and *L*_4_ − *L*_1_ remain nearly constant, without a notable long-term drift, within 12.8 nm and 7.1 nm in terms of 1,000-point averaging, respectively. When the differences are evaluated in terms of the Allan deviation as shown in Fig. 6c, the stability is 3.4 nm and 4.0 nm at 1 s averaging with a fractional stability of 8.9 × 10^−10^ and 1.1 × 10^−9^, respectively. The small discrepancy between *L*_3_ − *L*_2_ and *L*_4_ − *L*_1_ is attributable to the phase detection error that is random and independent of wavelengths as discussed in the next section of uncertainty evaluation. The stability reaches 0.57 nm and 0.78 nm at 100 s averaging, respectively, which corresponds to 1.5 × 10^−10^ and 2.1 × 10^−10^ of fractional stability. This result implies that even though the Ciddor’s formula is limited to a 10^−8^ level of uncertainty in estimating air refractive indices, the stability of distance measurement can be achieved better by about two orders of magnitude by properly adjusting the averaging time. However, if the averaging time is taken unnecessarily long, the measurement stability weakens due to the long-term drift of the environment monitoring sensors and also the phase-detection electronics.

### Uncertainty evaluation

Table 1 lists the uncertainty evaluation made for the multi-wavelength interferometry described so far. As derived in [ref. 16], the uncertainty *u*(*L*) of the distance measured by optical wave-length interferometry is contributed by three major terms; *u*(*L*) = [{*u*(*e*) × (λ/2)}^2^ + {*u*(*n*) × *L*}^2^ + {*u*(*f*) × *L*}^2^]^1/2^. The first term represents the electronic error with *u*(*e*) being the uncertainty of phase detection. Using a well-calibrated function generator, the multi-channel phase meter used in our interferometer system was assessed to have a maximum random error of 0.3° over the entire 360° range, i.e. *u*(*e*) = ~0.00083, bringing about a 0.64 nm distance error. This phase-induced error is not affected by increasing *L*, thus the least significant except when measuring short distances with sub-nm resolutions. The next important contribution is concerned with the frequency uncertainty *u*(*f*) of the light source. The random error caused by the frequency instability was estimated to be ~2.57 × 10^−11^ at 1 s averaging as discussed in the previous section. In addition, the systematic frequency offset of the Rb clock was provided by the manufacturer to be 5.00 × 10^−11^ as listed in Table 1. Thus, by combining the random error with the systematic offset, the frequency uncertainty *u*(*f*) is calculated as 5.63 × 10^−11^. This implies that the comb-reference wavelength generation performed in this work makes the frequency uncertainty causes no larger errors than the previous phase error until the measured distance *L* exceeds hundreds of meters.

It is now obvious that the most dominant contribution comes from the uncertainty *u*(*n*) related to the refractive index of air. This environment-dependent uncertainty has two distinct error sources; one is the inaccuracy arising in monitoring the ambient conditions and the other is the uncertainty of the dispersion formulas available for the air. The Ciddor’s equation or updated Edlen’s equation is deliberated to offer a 10^−8^ level uncertainty due to empirical-analytical limitations. In this work, adopting the state-of-the-art precision sensors permitted observing the ambient temperature within an uncertainty of 5 mK, the air pressure within 2.5 Pa, the air humidity within 1% and the carbon dioxide con-centration within 41 ppm. This precision enabled compensation of the refractive index of air to the extent of making the most of the 10^−8^ uncertainty, leaving the air dispersion formulas as the ultimate limit. In consequence, the combined uncertainty (*k* = 1) of the measured distance *L* is worked out to be *u*(*L*) = [(0.64 nm)^2^ + (1.62 × 10^−8^ × *L*)^2^]^1/2^, which estimates a total error of 62 nm for our experiments performed over a target distance of 3.8 m.

**Figure 7:**
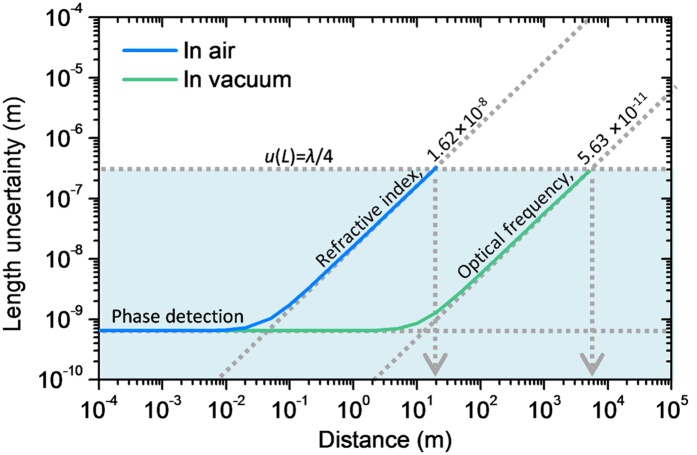
Uncertainty as a function of the distance to be measured in air. The λ/4-uncertainty limit line for multi-wavelength interferometry is shown to estimate the maximum measureable distance in air and vacuum.

Another important issue related to the uncertainty evaluation is the process reliability of multi-wavelength interferometry for absolute distance measurement. As discussed in the previous section, convergence to the true solution of *L* is guaranteed only when the uncertainty is less than a quarter of λ, i.e. *u*(*L*) < λ/4. This condition implies that the maximum measurable distance *L*_*max*_ is constrained as *u*(*L*) scales with *L* as depicted in Fig. 7. Based on the uncertainty data given in Table 1, *L*_*max*_ in air corresponds to 23.6 m for a 68% level of confidence (*k* = 1), being limited mainly by the uncertainty of estimating the refractive index of air. On the other hand, *L*_*max*_ in vacuum extends to 6.8 km as it is affected only by the uncertainty of the light source frequency. This reasoning supports the potential of the comb-referenced multi-wavelength interferometry in outer space use for various applications such as multiple satellites operation in formation^41^. Further improvements in the uncertainty of the source frequency may be made by several orders of magnitudes by stabilizing the reference comb to well-certified optical clocks^42^.

## Discussions

The multi-wavelength interferometer proposed and tested in this study is found capable of performing distance measurements with several fundamental advantages over conventional laser interferometers. First, the wavelengths generated with reference to the comb being stabilized to the Rb clock are highly stable and also accurate within an uncertainty level of 10^−11^, which is not readily achievable by traditional lasers used in distance interferometry. Second, the comb-referenced wavelength generation allows for reliable absolute distance measurements with a minimum number of wavelengths optimally selected for an extended non-ambiguity range well suited for precision machine-axis control and length metrology. Third, a comprehensive uncertainty evaluation can be made on the overall measurement performance with systematic identification of optical, electrical and environmental error sources.

The experimental work conducted in this study by measuring distances up to 3.8 m demonstrates a 0.57 nm repeatability and a 10 nm linearity, equivalent to 1.5 × 10^−10^ and 2.6 × 10^−9^ in fractional terms, respectively. The combined measurement uncertainty is estimated to be 1.62 × 10^−8^, which is mainly contributed by the ambiguity in estimating the refractive index of the ambient air. In vacuum environment, the measurement uncertainty is expected to improve to a 10^−10^ level which is accurate, stable enough for mass-production of optoelectronic devices with direct traceability to the atomic clock. It is also anticipated that the proposed method could be exploited for outer space scientific applications such as gravitation wave detection in the near future with further improvements on frequency stabilization by adopting optical clocks and also interferometric phase detection by incorporating high performance electronics.

## Additional Information

**How to cite this article**: Jang, Y.-S. *et al*. Comb-referenced laser distance interferometer for industrial nanotechnology. *Sci. Rep*. **6**, 31770; doi: 10.1038/srep31770 (2016).

## Figures and Tables

**Figure 1:**
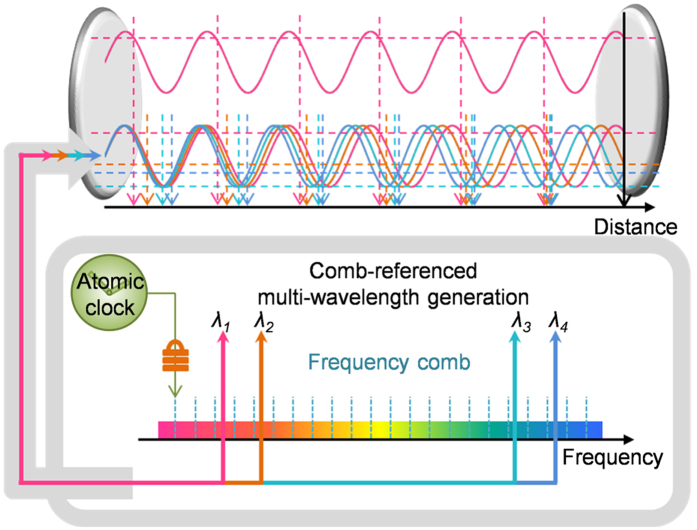
Comb-referenced multi-wavelength interferometry for realization of distance measurement with improved uncertainty evaluation.

**Table 1:** Uncertainty evaluation of distance measurement.

Uncertainty sources	Uncertainty
Uncertainty for phase detection	8.34 × 10^−4^ × λ/2 (0.64 nm)
Phase offset (systematic)	8.33 × 10^−4^ × λ/2
Repeatability (random)	2.94 × 10^−5^ × λ/2
Uncertainty for refractive index	1.62 × 10^−8^ × *L*
Temperature of air (ΔT = 5 mK)	4.56 × 10^−9^ × *L*
Pressure of air (ΔP = 2.5 Pa)	6.52 × 10^−9^ × *L*
Humidity of air (ΔH = 1%)	8.79 × 10^−9^ × *L*
CO_2_ concentration (ΔP_CO2_ = 41 ppm)	4.67 × 10^−9^ × *L*
Ciddor’s formula	1.00 × 10^−8^ × *L*
Uncertainty for frequency	5.63 × 10^−11^ × *L*
Frequency offset of *f*_*Rb*_ (systematic)	5.00 × 10^−11^ × *L*
Stability of *f*_*Rb*_ (random)	2.57 × 10^−11^ × *L*
Stability of *f*_*r*_(random)	3.08 × 10^−12^ × *L*
Stability of *f*_*ceo*_(random)	1.16 × 10^−14^ × *L*
Stability of *f*_*b*_(random)	8.49 × 10^−14^ × *L*
Combined standard uncertainty (*k* = 1)	[(0.64 nm)^2^ + (1.62 × 10^−8^ × *L*)^2^]^1/2^

## References

[b1] Kim S.-W. Metrology: combs rule. Nat. Photon. 3(6), 313–314 (2009).

[b2] Berkovic G. & Shafir E. Optical methods for distance and displacement measurements. Adv. Opt. Photon. 4(4), 441–473 (2012).

[b3] Gao W. . Measurement technologies for precision positioning. CIRP Ann. Manuf. Technol. 64(2), 773–796 (2015).

[b4] Bobroff N. Recent advances in displacement measuring interferometry. Meas. Sci. Technol. 4(9), 907–926 (1993).

[b5] Giacomo P. News from the BIPM. Metrologia 20(1), 25–30 (1984).

[b6] Quinn T. J. Practical realization of the definition of the metre, including recommended radiations of other optical frequency standards (2001). Metrologia 40(2), 103–133 (2003).

[b7] Felder R. Practical realization of the definition of the metre, including recommended radiations of other optical frequency standards (2003). Metrologia 42(4), 323–325 (2005).

[b8] Diddams S. A., Jones D. J., Ye J., Cundiff S. T. & Hall J. L. Direct link between microwave and optical frequencies with a 300 THz femtosecond laser comb. Phys. Rev. Lett. 84(22), 5102–5105 (2000). 10990877 10.1103/PhysRevLett.84.5102

[b9] Udem Th., Holzwarth R. & Hänsch T. W. Optical frequency metrology. Nature 416(6877), 233–237 (2002). 11894107 10.1038/416233a

[b10] Diddams S. A. . An optical clock based on a single trapped ^199^Hg^+^ ion. Science 293(5531), 825–828 (2001). 11452082 10.1126/science.1061171

[b11] Chun B. J., Hyun S., Kim S., Kim S.-W. & Kim Y.-J. Frequency-comb-referenced multi-channel fiber laser for DWDM communication. Opt. Express 21(24), 29179–29185 (2013). 24514469 10.1364/OE.21.029179

[b12] Kim Y.-J., Kim Y., Chun B. J., Hyun S. & Kim S.-W. All-fiber-based optical frequency generation from an Er-doped fiber femtosecond laser. Opt. Express 17(13), 10939–10945 (2009). 19550493 10.1364/oe.17.010939

[b13] Jin J., Kim Y.-J., Kim Y., Kim S.-W. & Kang C.-S. Absolute length calibration of gauge blocks using optical comb of a femtosecond pulse laser. Opt. Express 14(13), 5968–5974 (2006). 19516767 10.1364/oe.14.005968

[b14] Schuhler N., Salvadé Y., Lévêque S., Dändliker R. & Holzwarth R. Frequency-comb-referenced two-wavelength source for absolute distance measurement. Opt. Letters 31(21), 3101–3103 (2006). 10.1364/ol.31.003101 17041648

[b15] Salvadé Y., Schuhler N., Lévêque S. & Le Floch S. High-accuracy absolute distance measurement using frequency comb referenced multiwavelength source. Appl. Opt. 47(14), 2715–2720 (2008). 18470268 10.1364/ao.47.002715

[b16] Hyun S., Kim Y.-J., Kim Y., Jin J. & Kim S.-W. Absolute length measurement with the frequency comb of a femtosecond laser. Meas. Sci. Technol. 20(9), 095302 (2009).

[b17] Hyun S., Kim Y.-J., Kim Y. & Kim S.-W. Absolute distance measurement using the frequency comb of a femtosecond Laser. CIRP Ann. Manuf. Technol. 59(1), 555–558 (2010).

[b18] Wang G. . Absolute positioning by multi-wavelength interferometry referenced to the frequency comb of a femtosecond laser. Opt. Express 23(7), 9121–9129 (2015). 25968746 10.1364/OE.23.009121

[b19] Wu G., Takahashi M., Arai K., Inaba H. & Minoshima K. Extremely high-accuracy correction of air refractive index using two-colour optical frequency combs. Sci. Rep. 3, 1894 (2013). 23719387 10.1038/srep01894 PMC3667570

[b20] Kang H. J., Chun B. J., Jang Y.-S., Kim Y.-J. & Kim S.-W. Real-time compensation of the refractive index of air in distance measurement. Opt. Express 23(20), 26377–26385 (2015). 26480151 10.1364/OE.23.026377

[b21] Minoshima K. & Matsumoto H. High-accuracy measurement of 240-m distance in an optical tunnel by use of a compact femtosecond laser. Appl.Opt. 69(30), 5512–5517 (2000). 18354548 10.1364/ao.39.005512

[b22] Doloca N. R., Meiners-Hagen K., Wdeed M., Pollinger F. & Abou-Zeid A. Absolute distance measurement system using a femtosecond laser as a modulator. Meas. Sci. Technol. 21(11), 115302 (2010).

[b23] Jang Y.-S. . Absolute distance measurement with extension of nonambiguity range using the frequency comb of a femtosecond laser. Opt. Eng. 53(12), 122403 (2014).

[b24] Joo K.-N. & Kim S.-W. Absolute distance measurement by dispersive interferometry using a femtosecond pulse laser. Opt. Express 14(13), 5954–5960 (2006). 19516765 10.1364/oe.14.005954

[b25] Joo K.-N., Kim Y. & Kim S.-W. Distance measurements by combined method based on a femtosecond pulse laser. Opt. Express 16(24), 19799–19806 (2008). 19030065 10.1364/oe.16.019799

[b26] van den Berg S. A., Persijn S. T., Kok G. J. P., Zeitouny M. G. & Bhattacharya N. Many-wavelength interferometry with thousands of lasers for absolute distance measurement. Phys. Rev. Lett. 108(18), 183901 (2012). 22681076 10.1103/PhysRevLett.108.183901

[b27] van den Berg S. A., van Eldik S. & Bhattacharya N. Mode-resolved frequency comb interferometry for high-accuracy long distance measurement. Sci. Rep. 5, 14661 (2015). 26419282 10.1038/srep14661 PMC4588503

[b28] Coddington I., Swann W. C., Nenadovic L. & Newbury N. R. Rapid and precise absolute distance measurements at long range. Nat. Photon. 3(6), 351–356 (2009).

[b29] Liu T.-A., Newbury N. R. & Coddington I. Sub-micron absolute distance measurements in sub-millisecond times with dual free-running femtosecond Er fiber-lasers. Opt. Express 19(19), 18501–18509 (2011). 21935219 10.1364/OE.19.018501

[b30] Lee J. . Absolute distance measurement by dual-comb interferometry with adjustable synthetic wavelength. Meas. Sci. Technol. 24(4), 045201 (2013).

[b31] Zhang H., Wei H., Wu X., Yang H. & Li Y. Absolute distance measurement by dual-comb nonlinear asynchronous optical sampling. Opt. Express 22(6), 6597–6604 (2014). 24664008 10.1364/OE.22.006597

[b32] Lee J., Kim Y.-J., Lee K., Lee S. & Kim S.-W. Time-of-flight measurement using femtosecond light pulses. Nat. Photon. 4(10), 716–720 (2010).

[b33] Lee J., Lee K., Lee S., Kim S.-W. & Kim Y.-J. High precision laser ranging by time-of-flight measurement of femtosecond pulses. Meas. Sci. Technol. 23(6), 065203 (2012).

[b34] Han S., Kim Y.-J. & Kim S.-W. Parallel determination of absolute distances to multiple targets by time-of-flight measurement using femtosecond light pulses. Opt. Express 23(20), 25874–25882 (2015). 26480101 10.1364/OE.23.025874

[b35] Jones D. J. . Carrier-envelope phase control of femtosecond mode-locked lasers and direct optical frequency synthesis. Science 288(5466), 635–639 (2000). 10784441 10.1126/science.288.5466.635

[b36] Kim Y., Kim S., Kim Y.-J., Hussein H. & Kim S.-W. Er-doped fiber frequency comb with mHz relative linewidth. Opt. Express 17(14), 11972–11977 (2009). 19582112 10.1364/oe.17.011972

[b37] Ciddor P. E. Refractive index of air: new equations for the visible and near infrared. Appl. Opt. 35(9), 1566–1573 (1996). 21085275 10.1364/AO.35.001566

[b38] Tsai M., Huang H., Itoh M. & Yatahai T. Fractional fringe order method using Fourier analysis for absolute measurement of block gauge thickness. Opt. Rev. 6(5), 449–454 (1999).

[b39] Falaggis K., Towers C. E. & Towers D. P. Method of excess fractions with applications to absolute distance metrology: analytical solution. Appl. Opt. 52(23), 5758–5765 (2013). 23938429 10.1364/AO.52.005758

[b40] Falaggis K., Towers D. P. & Towers C. E. Algebraic solution for phase unwrapping problems in multiwavelength interferometry. Appl. Opt. 53(17), 3737–3747 (2014). 24921139 10.1364/AO.53.003737

[b41] Danzmann K. & Rüdiger A. LISA technology–concept, status, prospects. Class. Quant. Grav. 20(10), S1–S9 (2003).

[b42] Ludlow A. D., Boyd M. M., Ye J., Peik E. & Schmidt P. O. Optical atomic clocks. Rev. Mod. Phys. 87(2), 637–701 (2015).

